# Fibroids: yet another example of disparity in health?

**DOI:** 10.1186/s12916-022-02528-5

**Published:** 2022-09-06

Providing rapid and effective reproductive and maternal health is a core hallmark of a well-functioning society. Whilst women comprise half of all people in society, the importance of healthcare aspects specific to them is often de-prioritised. Following the recent anniversary of Fibroid Awareness Month in July, we focus on this under-discussed disorder, female reproductive health and why further action is needed to improve outcomes.

Female reproductive health encompasses healthcare and research related to the specific issues women face over the course of a lifetime. Dysfunction in the interplay of female reproductive organs and complex hormonal expression rhythms can cause a variety of disorders only experienced by women. Cancer of the Cervix, ovaries and endometrium, heavy menstrual bleeds, Uterine disorders, pre-, peri- and post-natal disorders are just a few examples of issues faced only by women. Fibroids are an example of a disorder faced by women with a challenging etiology and potential long term impact on quality of life.

Fibroids or Leiomyomas are benign smooth muscle tumours which form spontaneously in the pelvic and lower extremity. Whilst extremely rare cases have been identified in men, the tumours are most frequently found in the uterus. These masses can vary in size, and some rare recent case studies have identified fibroid masses larger than 5kg. In many cases, women with fibroids are asymptomatic. However, fibroids can cause abdominal or lower back pain, constipation, increased urination, pain during intercourse, heavy and/or painful periods and potential pregnancy complications or infertility, and thus can severely affect a woman's quality of life. The severity of symptoms varies and depends on the uterine location, number and size of fibroids present. Fibroids located submucosally, which then distort and invade the womb, are of particular concern for future pregnancies and fertility.

Startlingly, by the age of 50, 70 - 80% of women will have personally experienced some form of uterine fibroid growth. Further solidifying the prevalence of fibroids, 171 million women globally were estimated to have chronic fibroid symptoms from the 2013 Global burden of Disease (GBD) study on chronic diseases. In 2019, 1,378,498 disability-adjusted life years were attributed to fibroids, as reported by The Institute for Health Metrics and Evaluation (IHME). This significant burden of disease prompted the organisation to declare July as Fibroid Awareness Month, under this year’s theme “Real solutions”. Why then, despite the awareness day and a demonstrable burden on health, are healthcare issues due to fibroids not more highly prioritized?

One of the major problems surrounding fibroids is the lack of clear understanding of the cause. The current primary hypothesis focuses on excess estrogen and progestogen, promoting fibroid growth and development. There also appears to be a genetic component as shown by the increased concordance of fibroids in monozygotic twins versus other sibling pairs. However, a single genetic locus responsible for a majority of fibroid cases has not been identified. Risk factors for fibroids include heredity, low Vitamin-D levels, lack of previous childbirth, obesity, consumption of red meat and alcohol, polycystic ovary syndrome, diabetes and hypertension.

A key risk factor for fibroids is ethnicity. A large amount of research demonstrates Black and Asian women develop fibroids earlier, in greater numbers and larger sizes than matched White women. The causes are unclear, but a number of hypotheses link general disparities experienced by these Black and Asian women to the risk factors discussed above. As with all disparities, targeted interventions, including screening and early treatment campaigns, are necessary to provide more equitable healthcare to minority groups.

A variety of treatment modalities exist for fibroids. In many instances, asymptomatic fibroids will recede without intervention. Post-menopausal decreases in estrogen levels also increase the likelihood of fibroid atrophy. Medical interventions may target symptoms alone. Hormones such as Levonorgestrel can help to reduce period pain and heavy bleeding. Gonadotropin-releasing hormone analogues reduce estrogen release and directly promote fibroid reduction. Surgical interventions may be utilised in severe cases. For example, myomectomies directly excise fibroids from the uterine walls. Of deep concern to women undergoing this procedure is the risk that fibroids will recur in 10-25% of cases, prompting repeat myomectomy surgery. In extreme instances, hysterectomies are employed, which involve the removal of the entire womb. The emotional and mental burden associated with recurring fibroids, repeat surgery or the complete loss of reproductive ability takes a huge toll. As such, Obstetricians and Gynecologists play a key role in the quality of care received. Addressing these lived with and potentially stigmatizing issues requires greater interdisciplinary collaboration. More than simply focusing on the role of Obstetricians and Gynecologists, the inclusion of mental health practitioners and if appropriate social care workers should be encouraged for women’s reproductive disorders.

Quality of care for women with fibroids is an important issue at three levels: Access to proper screening and diagnosis, the availability of treatment modalities and pre- and post-treatment support and wellbeing guidance. Screening for fibroids is essential as a lack of awareness of the disorder can lead to many women suffering in silence from what may seem to be “typical” reproductive pains. In regions or countries where female and reproductive health is not prioritised, access to different modalities of treatment for fibroids may not be offered. In many areas, lack of funding for reproductive health or poor provider knowledge can lead to extreme treatment options such as hysterectomies being utilised as first-line treatment. These differences can be observed in resource-rich settings with healthcare providers refusing to provide the most appropriate levels of care and in resource-poor settings where women’s health is not prioritised. Patient cooperation should be at the heart of treatment options offered, ensuring the individual is able to lead a full and healthy reproductive life. In turn, greater support for women and families living with fibroids in terms of providing information on causes, potential lifestyle changes, how to handle setbacks, why diagnosis can be difficult during the perinatal period and all aspects related to their mental health is critical.

Sustainable development goal 3 (Ensure healthy lives and promote well-being for all at all ages) which targets 2030, focuses on reducing maternal mortality and providing universal access to sexual and reproductive health. In line with this goal, tackling the issues related to fibroid awareness and treatment should be a priority at all levels of healthcare provision. At a time when women’s health and reproductive rights are being undermined in resource-rich and poor regions, our healthcare systems must meet the specific needs of women. These interventions should also be balanced with a particular focus on ethnic disparities regarding prevalence and severity of fibroids. Alongside this, it is clear that specific research funding prioritisation is required to understand the causes of this disorder more clearly. At present, there is a lack of knowledge on how best to prevent fibroid formation, which limits our ability to reduce the global prevalence of the disorder. If we are serious about providing equitable healthcare to all members of society, we must prioritise issues specific to women.

